# A systematic review of methods for specifying the target difference in randomised controlled trials (delta review)

**DOI:** 10.1186/1745-6215-14-S1-O122

**Published:** 2013-11-29

**Authors:** Jonathan Cook, Jenni Hislop, Temitope Adewuyi, Kirsten Harrild, Cynthia Fraser, Doug Altman, Craig Ramsay, Peter Fraser, Andrew Briggs, John Norrie, Ian Harvey, Brian Buckley, Luke Vale

**Affiliations:** 1 University of Aberdeen, Aberdeen, UK; 2 Newcastle University, Newcastle upon Tyne, UK; 3 University of Oxford, Oxford, UK; 4 University of Glasgow, Glasgow, UK; 5 University of East Anglia, Norwich, UK; 6 National University of Ireland, Galway, UK

## Background

Determining the sample size is a vital aspect of randomised control trial design; typically a (target) difference is specified. This provides reassurance that the study will be informative; i.e. should such a difference exist, it is likely to be detected with the required statistical precision. From both a scientific and ethical standpoint, selecting an appropriate target difference is of crucial importance; too large or small a study is arguable unethical, wasteful and potentially misleading. While a variety of methods have been proposed to specify a target difference, their relative merits are unclear.

## Aim

To review systematically medical and non-medical literature to identify methods for specifying the target difference in a randomised controlled trial.

## Methods

Electronic searches of medical and non-medical databases were performed. Clinical trial textbooks were also reviewed. Titles and abstracts were screened prior to full-text assessment. Studies that reported a method that could be used to specify an important and/or realistic difference were included.

## Results

The search identified 11,485 potentially relevant studies; 1,434 were selected for full-text assessment. Seven methods were identified: anchor, distribution, health economic, opinion-seeking, pilot study, reviews of the evidence base and standardised effect size (SES). The anchor, distribution and SES methods were most commonly used.

## Discussion

Seven methods, each with important variations in application and different advantages and disadvantages are available to inform specification of the target difference. Their use can help ensure future trials will provide a meaningful finding and be an efficient use of resources.

